# The Progression of Mycosis Fungoides During Treatment with Mogamulizumab: A BIO-MUSE Case Study of the Tumor and Immune Response in Peripheral Blood and Tissue

**DOI:** 10.3390/biomedicines13010186

**Published:** 2025-01-14

**Authors:** Angelica Johansson, Eirini Kalliara, Emma Belfrage, Teodor Alling, Paul Theodor Pyl, Anna Sandström Gerdtsson, Urban Gullberg, Anna Porwit, Kristina Drott, Sara Ek

**Affiliations:** 1 Department of Immunotechnology, Faculty of Engineering (LTH), Lund University, 223 63 Lund, Sweden; 2 Department of Dermatology and Venereology, Skane University Hospital (SUS), 205 02 Lund, Sweden; 3 Department of Laboratory Medicine, National Bioinformatics Infrastructure Sweden, Science for Life Laboratory, Lund University, 221 00 Lund, Sweden; 4 Department of Laboratory Medicine, Lund University, 221 00 Lund, Sweden; 5 Division of Pathology, Department of Clinical Sciences, 221 00 Lund, Sweden; 6 Division of Medical Oncology, Department of Clinical Sciences, 221 00 Lund, Sweden

**Keywords:** biomarker discovery, cutaneous T-cell lymphoma, mycosis fungoides, patient prognostication, personalized medicine, spatially resolved transcriptomics, scRNA-seq, TCR sequencing

## Abstract

**Background/objectives:** Mycosis fungoides (MF) is a rare malignancy, with an indolent course in the early stages of the disease. However, due to major molecular and clinical heterogeneity, patients at an advanced stage of the disease have variable responses to treatment and considerably reduced life expectancy. Today, there is a lack of specific markers for the progression from early to advanced stages of the disease. To address these challenges, the non-interventional BIO-MUSE trial was initiated. Here, we report on a case study involving one patient, where combined omics analysis of tissue and blood was used to reveal the unique molecular features associated with the progression of the disease. **Methods:** We applied 10× genomics-based single-cell RNA sequencing to CD3+ peripheral T-cells, combined with T-cell receptor sequencing, to samples collected at multiple timepoints during the progression of the disease. In addition, GeoMx-based digital spatial profiling of T-helper (CD3+/CD8−), T-cytotoxic (CD3+/CD8+), and CD163+ cells was performed on skin biopsies. **Results.** The results pinpoint targets, such as transforming growth factor β1, as some of the mechanisms underlying disease progression, which may have the potential to improve patient prognostication and the development of precision medicine efforts. **Conclusions:** We propose that in patients with MF, the evolution of the malignant clone and the associated immune response need to be studied jointly to define relevant strategies for intervention.

## 1. Introduction

Cutaneous T-cell lymphoma (CTCL) is a rare, lymphoproliferative malignancy that infiltrates the skin [1]. Despite the indolent nature of the disease and that most patients have normal life expectancy, CTCL patients have highly heterogeneous clinical, histopathological, and immunophenotypic characteristics [2,3,4,5]. Mycosis fungoides (MF) is the most frequent subtype and comprises 60% of CTCLs, while the rarer Sézary syndrome (SS) subtype accounts for 5% of CTCL cases [6,7]. MF and SS are regarded clinically and pathologically as two closely related diseases that arise from mature skin-resident or skin-homing CD4+ T lymphocytes [8]. They are characterized by clonal, proliferative CD4+ T-cells, which in MF are mainly localized in the skin, while in SS, they are found circulating in peripheral blood [9]. CTCL has an incidence of 6–9 cases per million people, a male predominance, with an approximate 2:1 male to female ratio, and a median age at diagnosis of 50–60 years [6,10,11]. The extent of the disease in the skin is measured according to the modified Severity-Weighted Assessment Tool (mSWAT) [12,13]. MF and SS are staged according to the TNMB (tumor node visceral blood) classification, assessing the involvement of the skin, peripheral lymph nodes, peripheral blood, and visceral organs [14]. In the early stages of MF (stage IA), skin involvement presents with limited patches or plaques that involve less than 10% of the affected body surface area (BSA), and patients have a favorable prognosis, with a 5-year overall survival (OS) rate of 94% [10,15]. Patients with generalized patches and plaques that involve more than 10% of their BSA (stage IB) still have a good prognosis, with a 5-year OS rate of 84%. However, in about 25% of early-stage patients, the disease progresses to an advanced stage of disease, with widespread patches and plaques, ulcerated tumors, erythroderma and blood/systemic involvement, and the 5-year OS rate is only 18% (stage IVB) [7,10]. 

The heterogeneity of the disease complicates efforts to identify relevant predictive biomarkers for disease progression. To allow personalized treatment, an improved understanding of the response to treatment for subtypes of the disease is warranted. The interplay between the tumor immune microenvironment (TIME) and the malignant cells impacts disease biology and progression, and it is hypothesized that targeted immunotherapy will improve patient outcomes. It is well known that malignant cells promote a shift in the patient’s immune profile from a T-helper 1 (Th1) to a T-helper 2 (Th2) dominant profile, supporting immune dysregulation and tumor growth [16]. Furthermore, subtypes of the disease with different degrees of infiltrating cells, such as M2-like macrophages, B-cells, and/or exhausted T-cells, have been linked to a difference in the phenotype of the malignant cells and outcome of disease [17]. With modern technology, the malignant clone can be more accurately defined and separated from expanded reactive T-cells in the TIME [1]. This will improve the understanding of the evolution of the malignant clone over time, and facilitate the need to identify strategies to identify and treat patients with high risk of progression more optimally.

To address the need for improved personalized treatment strategies and risk management, a clinical trial at Skåne University Hospital, called “Predictive and prognostic biomarkers in patients with mycosis fungoides and Sézary syndrome”, i.e., the BIO-MUSE trial, was initiated [18]. Patients enrolled in the BIO-MUSE trial are monitored over time at three-month intervals, which includes sampling of blood and skin tissue, with the intention of capturing the progression-related transformational events of the disease. Here, we report on the first case study, involving a patient that experienced disease progression under treatment with mogamulizumab [19], targeting CCR4, and where the changes in the circulating malignant clone in the peripheral blood, as well as the changes in the TIME in skin biopsies, could be captured by comparing the biological features of the samples at different timepoints. Due to suspected side effects and disease progression, the patient was transferred to brentuximab vedotin (a drug conjugate targeting CD30) treatment in combination with CHEP (cyclophosphamide–doxorubicin–etoposide–prednisone). Our study pinpoints that progression of MF is driven by a single clonotype, but that multiple and equally expanded clonotypes are present at the steady-state disease and complicate the identification of the disease-driving malignant clone in early-stage disease. In this study, we show that previously identified MF-related features, such as expression of TOX and GATA3, are further enhanced in tissue as the patient progress and that the progression-associated clonotype specifically up-regulates transforming growth factor β1 (TGF-β1) prior to progression, which may be of interest to further investigate for future targeted treatment in sub-groups of MF patients.

## 2. Materials and Methods

### 2.1. Patient Material

**Figure 1:**
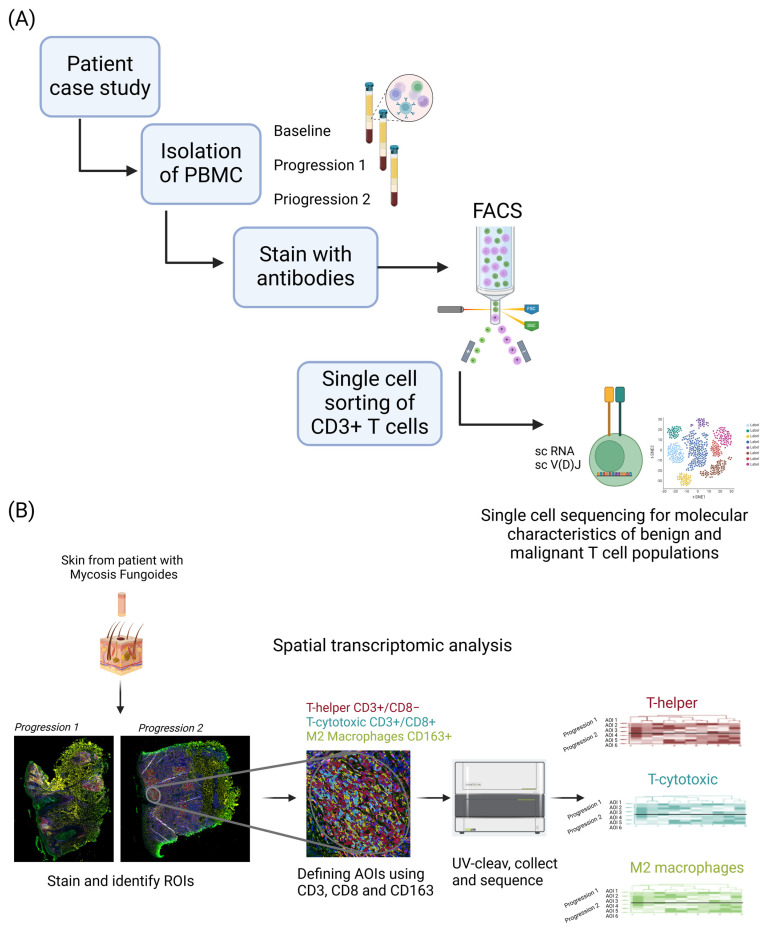
Study overview. Combined scRNA sequencing of peripheral blood and spatial omic profiling of tissue across time provide a global view of progression in a patient with cutaneous T-cell lymphoma. (**A**) For scRNA sequencing, combined 10× genomics-based 5’and TCRα/β sequencing workflows were used. (**B**) For spatial omic analysis, the digital spatial profiling GeoMx platform was applied.

The BIO-MUSE study is a prospective translational study registered with clinicalTrials.gov (NCT4904146). The clinical study was approved by the Swedish Ethical Review Authority (2019-05130). Informed consent was obtained from all patients before inclusion to the clinical trial. Inclusion criteria for participation in the study are age between 18 and 100 years, confirmed MF or SS diagnosis with stage IA-IVB, and World Health Organization performance status 0–3. The first patient was enrolled in April 2021 and the inclusion is ongoing, with 19 patients enrolled at the end of September 2024. Further details about the BIO-MUSE study design are available in the published study protocol [18]. The human peripheral blood samples used in this study were derived from one patient. Peripheral blood samples were collected at three different time points. Baseline corresponded to the peripheral blood sample collected and processed at the time of inclusion of the patient to the clinical trial. Progression 1 corresponded to the sample collected two months after initial patient inclusion when primary disease progression was confirmed. Progression 2 corresponded to the sample collected seven months upon initial patient inclusion when secondary disease progression was confirmed. Skin biopsies analyzed in this study were derived from the same time points, at progression 1 and progression 2. Clinical information on the case study patient is available in Table 1 and Supplementary Materials Table S1. An overview of the study is shown in Figure 1.

### 2.2. Clinicopathological Parameters

Treatment was performed according to clinical routine. Patient treatment included, for example, mogamulizumab, brentuximab–vedotin, and CHEP. Information on specific treatments at the different time points is shown in Table 1.

### 2.3. Isolation of Peripheral Blood Mononuclear Cells (PBMCs)

PBMCs were isolated from fresh peripheral blood samples by density gradient centrifugation over Ficoll-Paque (Cytiva, Marlborough, MA, USA). Briefly, peripheral blood samples were diluted in an equal volume of phosphate-buffered saline (PBS) solution (Cytiva SH30028.02) and transferred to a tube containing Ficoll-Paque. Isolated PBMCs were counted using the automated cell counter LUNA-FL^TM^ (Logos Biosystems, Logos Biosystems, Gyeonggi-do, Republic of Korea) and viability dye exclusion test was performed using Trypan blue 0.4% stain solution (Gibco, Thermo Fisher Scientific, Waltham, MA, USA). PBMCs at a concentration of approximately 5 × 10^6^ cells/mL were resuspended in RPMI-1640 media (Cytiva) supplemented with 20% fetal bovine serum (FBS, Gibco) and 10% dimethyl sulfoxide (DMSO, Sigma-Aldrich, Burlington, MA, USA) and cryopreserved in liquid nitrogen until further use.

### 2.4. Flow-Cytometry-Based Analysis

After thawing PBMCs, a total of 1 × 10^6^ cells were re-suspended in MACS buffer containing 0.5% bovine serum albumin (BSA, Cohn fraction V) and 200 mM ethylenediaminetetraacetic acid (EDTA, Thermo Fisher Scientific) in PBS. Viability staining was performed in 1:1000 dilution in PBS using BD Horizon fixable viability staining dye 780 (BD Biosciences, Franklin Lakes, NJ, USA) for 20 min at 4 °C in the dark, followed by incubation with Fc-blocking buffer (ChromePure Mouse IgG, Thermo Fisher Scientific) for 15 min at RT. After blocking, PBMCs were stained with fluorescently conjugated antibodies. All antibodies were purchased from BD Biosciences and are listed in Supplementary Materials Table S2. Antibodies were added, and incubation was performed for 20 min at 4 °C in the dark. PBMCs were stained with BV786 mouse anti-human CD3 and PE-Cy5 mouse anti-human CD56 for scRNA-seq sorting. Sample recording, analysis, and single-cell sorting were performed using a fluorescence-activated cell sorting (FACS) Aria II cell sorter (BD Biosciences) and the FACSDiva 9.0 software (BD Biosciences). Live cells were selected based on exclusion of the viability dye and CD3+ T-cells were selected as CD3+CD56− T-cells. A representative gating strategy is shown in Supplementary Materials Figure S1. Viable CD3+ T-cells were sorted FACS into ice-cold PBS containing 0.04% BSA.

### 2.5. Single Cell 5′ RNA and V(D)J Sequencing

FACS-sorted single CD3+ T-cells were loaded onto a 10× Genomics Chromium Next GEM Chip (10× Genomics, Pleasanton, CA, USA) using the 10× Genomics Chromium Next GEM Single Cell 5′ kit v2 and the Chromium Single Cell Human TCR Amplification Kit (10× Genomics) according to the manufacturer’s instructions. The number of sequenced cells in each sample is provided in Supplementary Materials Figure S2. Single Cell 5′ RNA and V(D)J libraries were subjected to NovaSeq 6000 Illumina sequencing (Illumina, San Diego, CA, USA) using a NovaSeq 6000 S1 reagent Kit v1.5 with 50,000 read pairs per cell for the Single Cell 5′ RNA Library and 10,000 read pairs per cell for the V(D)J/TCR library. Library preparation and sequencing was performed by the Centre for Translational Genomics (CTG) at Lund University. Pre-processing of raw sequencing data including reads alignment, filtering, barcode counting, and UMI counting on the Single Cell 5′ RNA library, as well as V(D)J transcript assembly and clonotype calling, was performed using the 10× Cell Ranger software (v. 7.0.0). Single Cell 5′ RNA reads were aligned to the Human genome reference (GRCh38), while V(D)J reads were aligned to the Human V(D)J reference (GRCh38). An overview of the scRNA-seq workflow is shown in Figure 1A.

#### 2.5.1. Single-Cell RNA Sequencing (scRNA-Seq) Data Processing

All downstream quality control (QC) and pre-filtering steps were performed using the Seurat (v. 4.4.0) R package. Poor-quality cells were removed by the following filter criteria: nFeature_RNA > 200, log10GenePerUMI (complexity score) > 0.8, mitochondrial genes less than 20%, and ribosomal genes more than 5%. After filtering cells of poor quality, a total of 5770 cells from the baseline sample, 1259 cells from the progression 1 sample, and 489 cells from the progression 2 sample were retained for downstream analysis (Supplementary Materials Table S3).

Post-filtering data were normalized and scaled using the SCTransform function and common anchors were identified to integrate and combine the three different samples and reduce technical variation across all libraries. Genes not found in more than three cells were removed. The top 2000 variable features were selected for principal component analysis (PCA), and the first 20 components were used for subsequent dimensionality reduction and downstream visualization. Cells were visualized using uniform manifold approximation and projection (UMAP). Unsupervised clustering was performed by utilizing a graph-based clustering approach. The first 20 principal components were used to construct the shared nearest neighbor (SNN) graph using the FindNeighbors function in Seurat. Subsequently cells were clustered by applying the FindClusters function, with a resolution set at 0.5, based on the Louvain algorithm to iteratively group cells together.

#### 2.5.2. Cell Annotation

To annotate cell clusters, a three-step workflow was followed, as previously recommended [20]. These included (a) automated cell annotation, (b) identification of top differentially expressed genes (DEGs) of each cluster, and (c) visualization of canonical/classical markers expression selected according to reference publications.

First, the R package SingleR (v. 2.4.1) [21] was utilized to annotate cell clusters in an automated manner by correlating query cell transcriptomic profiles with average reference profiles to infer the cell origin within each cluster of our dataset. Here, the package celldex (v. 1.12.0) was used to load the MonacoImmuneData reference dataset, which includes bulk-RNA-seq data of sorted human immune cells derived from 114 samples [22].

Second, differentially expressed genes for each cluster were identified using the Wilcoxon rank sum method with the FindAllMarkers Seurat function, where the logFC threshold was set at 0.2 and statistically significant differentially expressed genes (DEGs) were filtered and ordered by adjusted *p*-value < 0.05. The top 25 expressed genes in each cluster were carefully reviewed and reference articles were used to infer the most reasonable cell annotation [23,24]. Third, canonical markers expressed in each cluster were additionally reviewed to validate and help re-assign cell labels when considered necessary. The selected canonical markers are presented in Supplementary Materials Table S4.

#### 2.5.3. TCR Clonotype Identification

The TCR sequencing analysis with Cell Ranger was performed on a per-sample basis. To match clonotypes between samples we used the identified TCR sequences to label clonotypes consistently in all three samples. The clonotype information was then attached to the Seurat object as a metadata column by mapping the cell barcodes. Cells lacking clonotype information were removed. In total, 3115 cells with clonotype information were available in the baseline sample, 888 cells in the progression 1 sample, and 328 cells in the progression 2 sample, and were used for downstream analysis. Clonotype proportions in each sample were calculated as number of cells with the specific clonotype divided by the total number of cells with clonotype information available in the sample.

### 2.6. Digital Spatial Profiling

#### 2.6.1. Multiplex Immunofluorescent (mIF) Staining

Formalin-fixed paraffin-embedded (FFPE) skin biopsies from the patient at progression 1 and progression 2 were collected. Tissue sections, 4 µm thick, were placed on glass slides (Superfrost® Plus, Epredia, Portsmouth, NH, USA). The slides were stained with fluorescent antibodies and scanned at 20× using the GeoMx^®^ DSP instrument (Nanostring, Bruker Spatial Biology, Seattle, WA, USA). The staining and optimization of each antibody was performed according to GeoMx protocols (https://university.nanostring.com, accessed on 15 January 2022). To enable visualization of cells of interest, the tissues were stained with CD3, CD163, and CD8 antibodies in combination with nuclear counterstain Syto13 (S7575, Invitrogen™, Thermo Fisher Scientific). Information about the antibodies used for the multiplex immunofluorescent staining is available in Supplementary Material Table S2.

#### 2.6.2. Region of Interest (ROI) Selection

The selection of ROIs was guided by the staining of CD3, CD8, and CD163, which mark T-helper cells (CD3+/CD8−), T-cytotoxic cells (CD3+/CD8+), and CD163+ macrophages, respectively, and focused on regions with malignant cells (CD3+/CD8−) (Figure 1B, overview of selection strategy). Within the ROIs, targeted mRNA expression was quantified in each of the targeted cell types. These sub-regions containing the specific cell type of interest within an ROI are technically called areas of illumination (AOI) but will be referred to as segments in the Results and Discussion sections. In our study, the cells of interest were T-helper cells (CD3+/CD8−), T-cytotoxic cells (CD3+/CD8+), and CD163+ macrophages. The summary of number of ROIs/AOIs collected for the patient is provided in Supplementary Materials Figure S2. All cell types could not be investigated in each ROI, due to absence and/or low number of cells of one or more cell types.

#### 2.6.3. Retrieval of Probes for Proteomic and Transcriptional Analyses

Collection and quantification of the targets in each AOI was performed according to Nanostring protocols. Briefly, oligos conjugated to bound reagents (1811 mRNA probes) were cleaved off by ultraviolet light and collected into individual wells on a microtiter plate, for separate profiling of each cell subset, in each AOI. Information on the Cancer Transcriptome Atlas (CTA) panel is available from Nanostring Inc., as well as one of our previous studies [25].

#### 2.6.4. Pre-Processing of GeoMx™ Data

The probeQC counts, which are the default read-out from the GeoMx experiment, were used as input to the StandR workflow [26] and analyzed using the SpatialExperiment (v.1.14.0) package in R. Gene filtering was performed according to the addPerROIQC function within the StandR workflow, resulting in 1811 expressed genes for biological exploration. At the sample level, we considered both the library size and AOI area, as suggested in the StandR workflow, to identify the low-quality AOIs. One sample (AOI) was filtered out using a library size threshold of 2000. The data were normalized using the cyclic loess method.

### 2.7. Gene Expression Analysis

Gene expression analysis was performed using R (v. 4.4.0) and R studio (v. 2024.04.0+735, R Foundation for Statistical Computing, Vienna, Austria).

#### 2.7.1. Identification of Differentially Expressed Genes in scRNA-Seq Dataset

Analysis and visualization of the scRNA-seq and TCRseq data were performed mainly using Seurat (v.4.4.0) and SeuratObject (v.5.0.1). For differential expression analysis, the functions FindMarkers and FindAllMarkers using Wilcoxon rank sum test in Seurat were used, the logFC threshold was set at 0.25, and statistically significant DEGs were filtered by Bonferroni-adjusted *p*-value < 0.05. Further visualization was conducted with packages VennDiagram (v.1.7.3) and ggplot2 (v.3.5.1). Gene set enrichment analysis (GSEA) was performed using the KEGG_medicus database and GO Biological Process ontology database with the package fgsea (v.1.30.0).

#### 2.7.2. Identification of Differentially Expressed Genes in Digital Spatial Profiling Dataset

The SpatialDecon workflow in R by Nanostring [27] was used for the cell deconvolution. The limma voom (v.3.60.1) package, with an adjusted *p*-value < 0.05 set as cut-off, was used for differential expression analysis. Gene set enrichment analysis (GSEA) was performed using the KEGG_medicus database and GO Biological Process ontology database with fgsea (v.1.30.0). R packages such as ggplot2 (3.5.1) and ComplexHeatmap2 (2.18.0) were used for data visualization.

## 3. Results

The studied samples derive from a male BIO-MUSE patient, 71 years old at the time of MF diagnosis. He was enrolled in the study eight years after initial diagnosis. Two months after enrollment, the first signs of progression were observed with transformed cells in an enlarged lymph node in left axilla (progression 1). At the time of progression 1, treatment with mogamulizumab had been ongoing for nine months and was continued due to good systemic control in blood and skin overall. After 14 months of treatment with mogamulizumab, the patient was admitted with acute renal failure, diarrheas, and progression in the skin on the scalp (progression 2). This was interpreted as intolerable side effects of mogamulizumab in combination with progression. At that time, treatment was changed to a CD30 antibody–drug conjugate brentuximab–vedotin together with CHEP See Table 1 for treatment regimens used at the different time points. However, the patient did not respond well and passed due to the disease two years after enrollment in the BIO-MUSE trial. Sampling was performed according to study protocol, including peripheral blood draws at three-month intervals, and skin biopsies were performed at progression 1 and at progression 2.

### 3.1. Single-Cell RNA Sequencing of Peripheral Blood Samples

#### 3.1.1. Flow-Cytometry-Assisted Cell Sorting and Initial Quality Control

**Figure 2:**
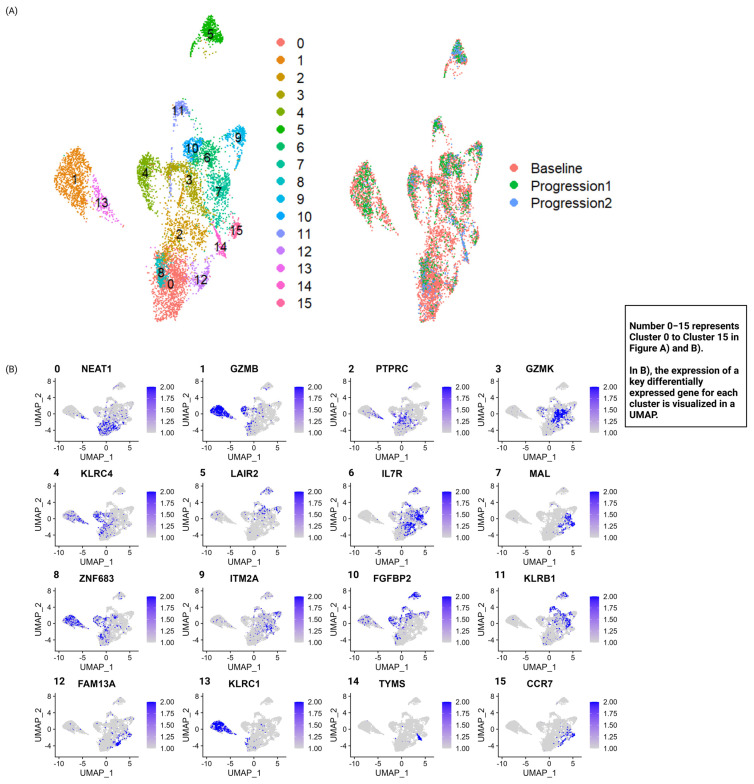
Transcriptionally distinct T-cell subsets. (**A**) CD3+ T-cells were separated into 16 distinct clusters through scRNA sequencing and are colored by the cluster number to the left, and by time point to the right. (**B**) One key differentially regulated gene per cluster, in total 16, is visualized in each feature plot and reveals biological differences between the clusters. The scale represents log-normalized expression values. Expression values below 1 and above 2 are truncated.

At least 10,000 cells were sorted, and went through preparatory steps, resulting in the sequencing of at least 1500 single cells from each time point. After QC, the number of cells evaluable varied from >5000 for the baseline sample, while ~1300 and ~500 cells were evaluable from the time of progression 1 and progression 2, respectively (Supplementary Materials Table S3). Using unsupervised clustering, with a resolution of 0.5 using the FindClusters function, 16 clusters could be identified and were visualized using uniform manifold approximation and projection (UMAP). The integrated UMAP showing cells from all timepoints is shown in Figure 2A, while the integrated UMAPs visualizing the individual time points are shown in Supplementary Materials Figure S3A. The top 25 differentially expressed genes (DEGs) for each cluster are listed in Supplementary Materials Table S5 and one of the top features for each cluster is visualized in Figure 2B, exemplifying the main biological difference between the identified clusters. Many markers are associated with important T-cell function, including expression of granzymes such as GZMB and K (cluster 1 and 3), killer lectin receptors C4, B1, and C1 (cluster 4, 11, and 13), the interleukin-7 receptor (cluster 6), and chemokine receptor 7 (cluster 15). Of note, other key differentially expressed genes have previously been associated with CTCL including LAIR2 [28] for cluster 5 and FGFBP2 [29] for cluster 10, emphasizing the biological relevance of the key DEGs.

#### 3.1.2. Cluster Identification Using Semi-Automated Method

**Figure 3:**
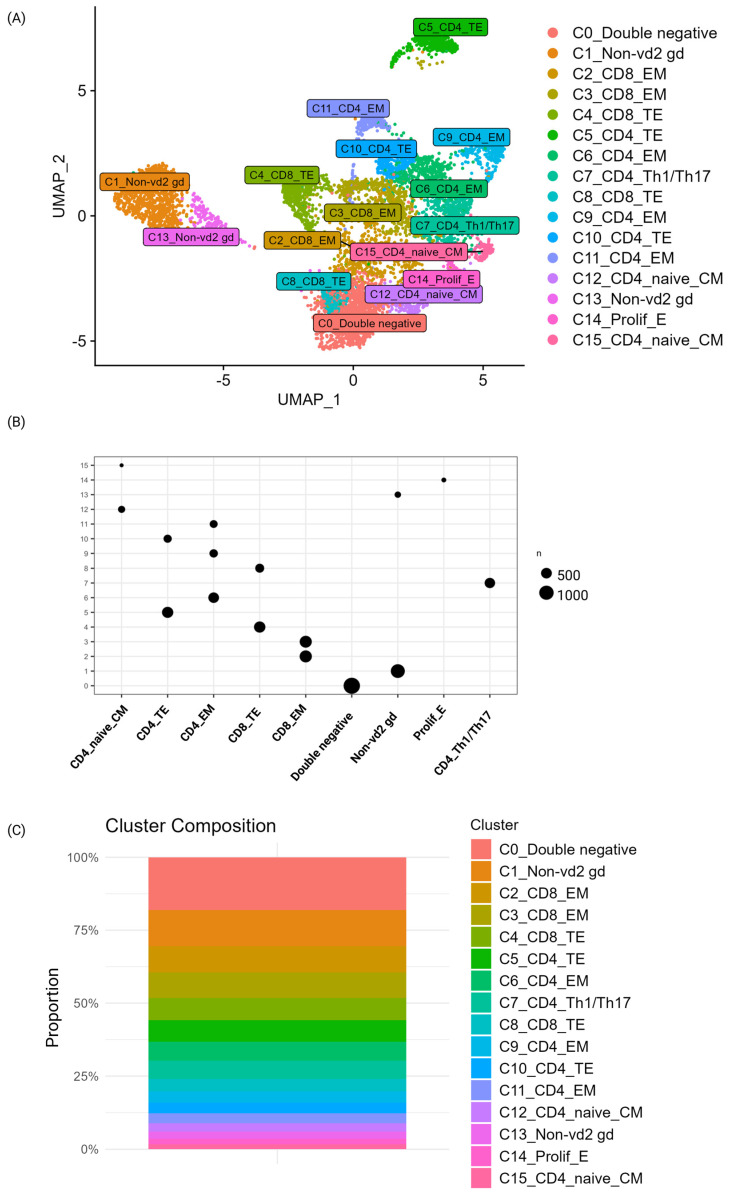
Cell classification of individual clusters. (**A**) A semi-automated approach was used to annotate the clusters, and nine phenotypically defined cell types were defined. The clusters are colored by the cluster and cell type label. (**B**) The number of total cells in the three time points in each cluster across the identified cell types. (**C**) The relative proportion of cells in the three time points in each cluster (0–15).

Cell type identification was performed using a semi-automated method where the SingleR workflow was applied, and manual validation of canonical phenotypic markers and of top DEGs (Supplementary Materials Table S5) was used to verify and complement the cell type identification for each cluster. The relative expression of the phenotypic markers used for verification are available in Supplementary Materials Figure S3B. The sixteen clusters were annotated, resulting in the identification of nine different cell types (Table 2 and Figure 3A). The number of cells in each of the clusters is shown across the defined cell types in Figure 3B and in Supplementary Materials Table S6. Both CD4- and CD8-positive T-cell populations were identified. Among CD4-positive cells, four subsets, including terminal effector, effector memory, Th1/Th17, and naïve/central memory T-cells, were identified. Among CD8-positive cells, two populations, including effector memory T-cells and terminal effector T-cells, were identified. Additional identified cell clusters included double-negative T-cells, non-vd2 gd, and proliferating effector T-cells. Surprisingly, no regulatory T-cell cluster was identified. The relative proportion of each cluster for the complete dataset with all three time points included is shown in Figure 3C and Supplementary Materials Table S6. The relative proportion of cells varied over time for many of the identified cell types, including proliferating effector T-cells that had more than 5× as many cells at progression 2 compared to earlier time points. Non-vd2 gd showed the opposite trend, with approximately one-third of the cell frequency at progression 2 compared to earlier time points. Visualizations of the relative proportions of each cell type at the three different timepoints are shown in Supplementary Materials Figure S4.

#### 3.1.3. Identification of Expanded Clonotypes Using T-Cell Receptor α/β Chain Sequencing

**Figure 4:**
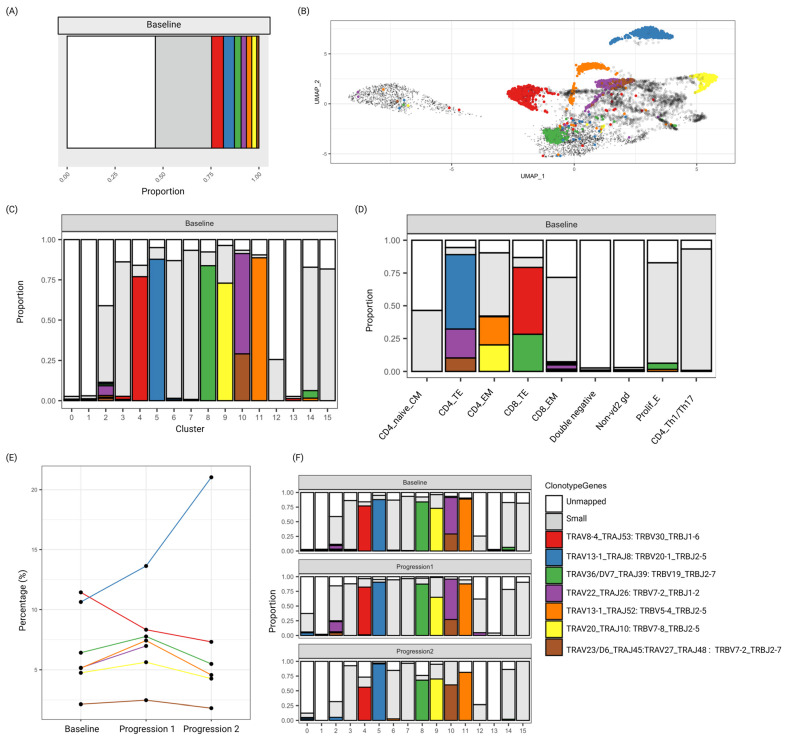
TCR sequencing reveals CD4+ and CD8+ expanded clones. The colors represent the clonotype genes indicated at the lower right panel. (**A**) The relative proportion of the top seven clonotypes among total number of cells at baseline displayed in one color each. Clonotypes only detected at a low proportion are jointly displayed in grey, and unmapped cells are displayed in white. (**B**) Clonotype identity of each cell in the integrated UMAP plot. The small black dots represent the unmapped cells with no T-cell receptor α/β chain. (**C**) The relative proportion of each of the top seven clonotypes across the clusters at baseline. (**D**) The relative proportion of each of the top seven clonotypes across the different cell types at baseline. (**E**) The percentage of TCR sequenced cells of each of the top seven clonotypes, visualized across the three time points (baseline, progression 1, and progression 2). (**F**) The relative proportion of the top seven clonotypes in each cluster, visualized across the three time points (baseline, progression 1, and progression 2).

TCR-αβ sequencing was performed, and clonotypes of in total 4149 cells were determined. The patient sample included several expanded clonotypes, and the top seven clones are visualized in Figure 4A. ScRNA-seq and clonotype data were combined to allow the mapping between expanded clones, clusters, and cell types. Analysis showed that most clonotypes had homogenous gene expression and were mapped to a single cluster (Figure 4B). Clusters 4, 5, 8, 9, 10, and 11 were dominated by a single clonotype, while the other clusters had only a small or no proportion of expanded cells (Figure 4C).

Each clonotype was further assigned to one of the cell types identified (Figure 4D). Such visualization revealed that the CD4 and CD8 terminal effector cells were oligoclonal. At baseline, the CD4 terminal effector cells were dominated by clonotypes TRAV13-1_TRAJ8 (60%), TRAV23/DV6_TRAJ45 (11%), and TRAV22_TRAJ26 (23%), which constituted 16% of the total TCR sequenced cells. For CD8 terminal effector cells, clonotypes TRAV8-4_TRAJ53 (59%) and TRAV36/DV7_TRAJ39 (32%) together constituted 17% of the successfully TCR sequenced cells. The CD4 effector memory cells included 20% of cells that were expanded from two distinct clonotypes (Figure 4D). All other cell types were polyclonal. As only TCR-αβ sequencing was performed, no TCR sequences were available for the γδ subset of T-cells.

The change in percentage (of TCR sequenced cells) of each clonotype over time was evaluated, and TRAV13-1_TRAJ8 showed a relative increase from baseline to progression 1, and from progression 1 to progression 2, and was therefore defined as a malignant clone, associated with disease progression (Figure 4E). The relative proportion of the identified expanded clonotypes at the three time points, visualized for each cluster, is shown in Figure 4F. The relative proportion of TRAV8-4_TRAJ53 decreased across time, which indicates that effective CD8 immunity was lost/reduced. Other time-related changes included the lack of TRAV22_TRAJ26 at progression 2. The relative increase over time of the progression-related malignant clonotype TRAV13-1_TRAJ8 among the CD4 terminal effector memory is shown in Supplementary Materials Figure S5. We hypothesize that all clonotypes with the same cell type label (CD4 terminal effector cells) are malignant cells. Thus, these cells would constitute TRAV13-1_TRAJ8 (C5, associated with disease progression), TRAV23/DV6_TRAJ45 (C10), and TRAV22_TRAJ26 (C10).

Analysis of CD7, which is known to be low in MF cells, was used to validate the assignment of C5/C10_CD4_TE cells as the two clusters harboring malignant cells. CD7 had significantly lower expression in both C5 and C10 compared to expanded CD4 (adjusted *p*-value was 2.8 × 10^−18^ for C5 and 3.6 × 10^−18^ for C10). The differential expression was assessed with Wilcoxon rank sum test with Bonferroni correction (Supplementary Materials Figure S6). There was no statistically significant difference in CD7 expression comparing other expanded to non-expanded CD4 clusters in the differential expression analysis.

#### 3.1.4. Molecular Features Associated with Identified Cell-Type-Specific Expanded Clonotypes

**Figure 5:**
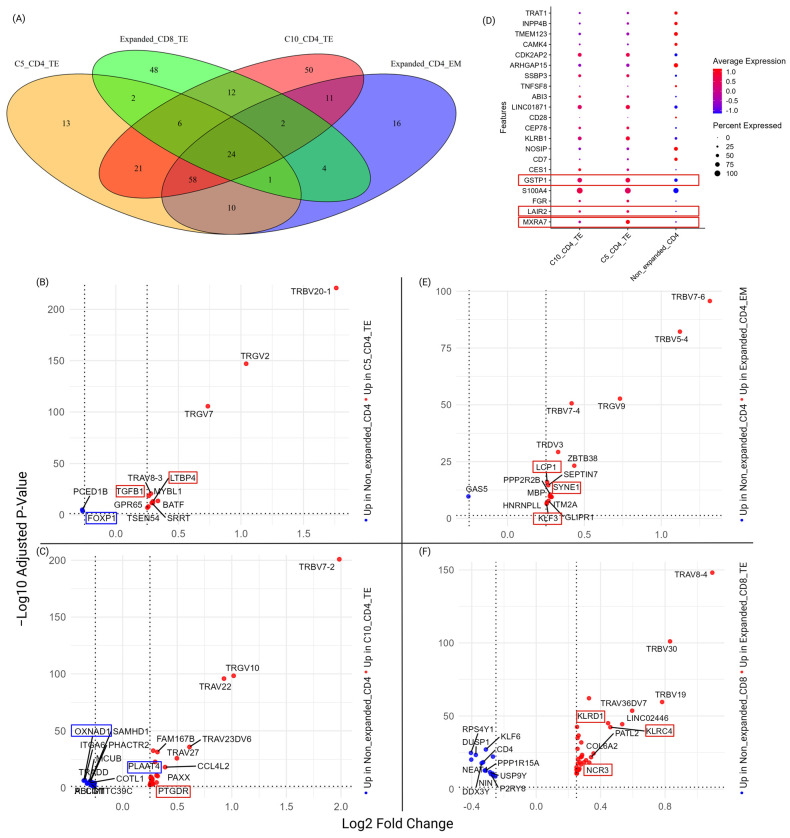
Differentially regulated genes (DEGs) at baseline. DEGs associated with different expanded compared to non-expanded populations were determined. (**A**) Venn diagram of unique and common genes across C5_CD4_TE, C10_CD4_TE, expanded CD4_EM, and expanded CD8_TE. (**B**) Volcano plot of the 13 unique DEGs associated with the progression-related C5_CD4_TE. (**C**) Volcano plot of the 50 unique DEGs associated with malignant population C10_CD4_TE. (**D**). Dot plot of the 21 common genes among C5_CD4_TE and C10_CD4_TE but that differ compared to non-expanded CD4 cells. Red boxes indicate key genes. (**E**) Volcano plot of the 16 unique DEGs associated with expanded CD4_EM cells. (**F**) Volcano plot of the 48 unique DEGs associated with expanded CD8_TE cells. Genes marked with red and blue boxes indicate important features for each expanded T-cell population.

To investigate the unique features associated with expanded clonotypes, DEGs for each of the expanded clusters (compared to non-expanded CD4 clusters C6, C7, C12, and C15) were identified (Supplementary Materials Figure S7A–D, Supplementary Materials Tables S7–S12). The number of common and unique DEGs are shown in Figure 5A. Beyond C5_CD4_TE (C5) and C10_CD4_TE (C10), expanded CD4 cells included clusters C9 and C11, while the expanded CD8 cells included clusters C4 and C8.

DEGs that were uniquely identified in the progression-related clone C5_CD4_TE are visualized in Figure 5B. Such C5-unique DEGs included several genes involved in the regulation of proliferation, such as MYBL1 and SRRT. Other up-regulated genes were BATF, which is essential for T-cell effector function and previously associated with MF [30] and GPR65, a member of the proton-activated G protein-coupled receptor (GPCR) family with pH sensing capability. Of note, both transforming growth factor β 1 (TGF- β1) and latent TGF- β1 binding protein (LTBP4), which control the activity of the former protein, were both uniquely overexpressed in C5. The association between TGF- β1 and progression of MF has not been reported before and is of clinical interest. Down-regulated genes included FOXP1 [31], important for regulation of T-cell activation, memory, and exhaustion (Figure 5B).

Unique DEGs were also identified for the malignant C10_CD4_TE cells (Figure 5C) and exemplify the heterogeneity among the different malignant clones. Here, for example, the prostaglandin D2 receptor (PTGDR) was up-regulated, while down-regulated genes were involved in apoptosis (TRADD), metabolism (OXNAD1), and regulation of proliferation, such as phospholipase γ 1 (PLCG1).

To further investigate the common molecular features of the malignant CD4_TE cells, the 21 common DEGs of C5 and C10 (Figure 5A) were visualized to emphasize the relative abundance and expression of the individual genes (Figure 5D) in the two clusters compared to non-expanded CD4 cells. Several of the identified genes have previously been involved in cancer drug resistance (Glutathione S-transferase Pi 1 (GSTP1)), remodeling of tissue (matrix remodeling associated 7 (MXRA7)), or interaction with extracellular matrix (the collagen receptor Leukocyte-Associated Immunoglobin-Like Receptor 2 (LAIR2)) [32].

Expanded CD4 T-cells, which may have been amplified due to infection or as a response to the tumor, were investigated by focusing on the 16 unique DEGS of the CD4_EM cells (Figure 5E, Supplementary Materials Table S12). Transcriptional up-regulation included genes coding for the cell cycle regulatory protein septin-7, the transcription factor Krüppel-like factor (KLF) 3 [33], actin binding lymphocyte cytosolic protein 1 (LCP1), and synaptic nuclear envelope protein (SYNE1).

It has previously been reported that the expansion of CD8 cells is important for the control of MF [34] and it is, thus, likely that expanded CD8 clonotypes are present in peripheral blood of patients. To investigate the molecular features of such expanded clones, C4/C8_CD8_TE were compared with non-expanded clones (C2/C3_CD8_EM), resulting in the identification of 48 unique DEGs that are shown in Figure 5F and Supplementary Materials Table S12. Up-regulated features in the expanded CD8 clones included genes associated with cytotoxicity, such as two killer cell lectin like receptors (KLRD1 and KLRC4) and natural cytotoxicity triggering receptor 3 (NCR3), indicating that such functions may be enhanced in the expanded cells compared to the non-expanded cells.

In conclusion, the analysis of expanded clones showed that DEGs specifically associated with the progression-related malignant cluster C5_CD4_TE could be identified. Novel genes associated with progression included TGF- β1 and LTBP4.

#### 3.1.5. Pathway Analysis of DEGs Associated with Expansion of T-Cell Clonotypes

**Figure 6:**
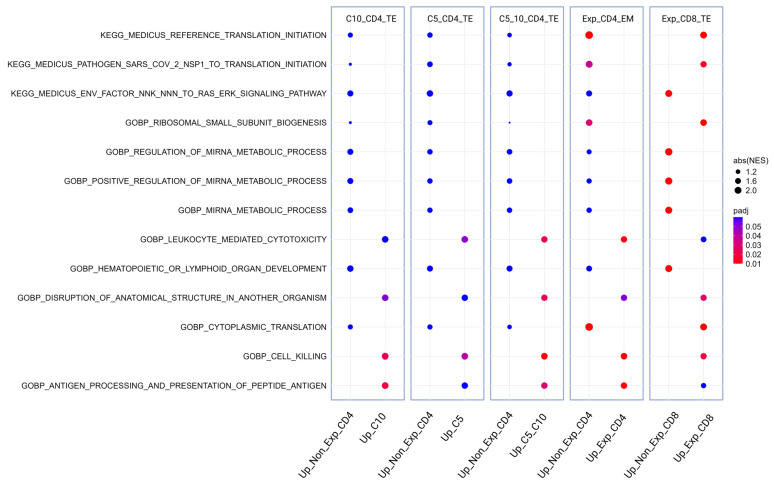
Pathway analysis comparing expanded peripheral T-cell populations compared to non-expanded cells. The top five identified pathways in each comparison driven by differentially regulated genes; the top significant (adjusted *p*-value < 0.05) pathways are ranked based on absolute normalized enrichment score (abs(NES)). The following comparisons were made: C5_C10, Exp_CD4 (C9 and C11), C10, and C5 were each compared to non-expanded CD4 cells (C6, C7, C12, C15). Exp_CD8 (C4 and C8) were compared to non-expanded CD8 cells (C2 and C3).

Pathway analysis was performed for each of the expanded cell populations (C5_CD4_TE and C10_CD4_TE, CD4_EM and CD8_TE) including a combined group with C5 and C10 (C5_C10_CD4_TE) compared to cells with non-expanded clonotypes (Non_expanded_CD4) (Figure 6). Expanded CD4_EM and expanded CD8_TE had more differentially regulated pathways compared to the malignant clones C5 and C10_CD4_TE, and several pathways were counter-regulated in expanded CD4 compared to expanded CD8 clusters. Of interest, on a related level, expanded CD4 cells seem to up-regulate cytotoxic features more than expanded CD8. Also, the combined analysis of malignant CD4 cells (C5_C10_CD4_TE) had similar up-regulation of “disruption of anatomical structure in another organisms” to expanded CD8. Cell killing was also enriched across all expanded populations. Of note, the two malignant clusters 5 and C10 showed a difference in the antigen processing and presentation of peptide antigen pathway. In contrast to C10, this pathway was not up-regulated significantly in the progression-related C5 cluster, indicating that reduced antigen presentation is an important feature of progression-related expanded sub-clones.

#### 3.1.6. Molecular Features Associated with the Progression of Disease

**Figure 7:**
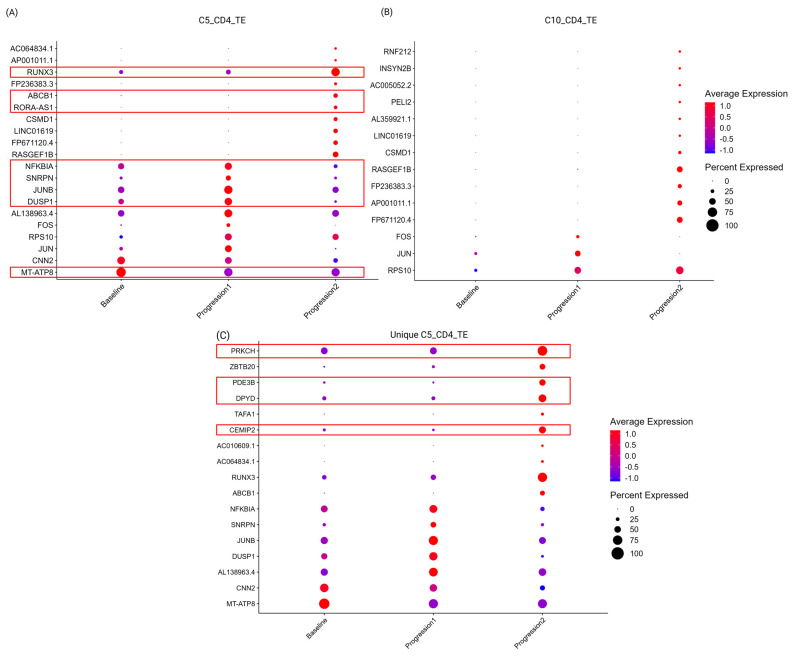

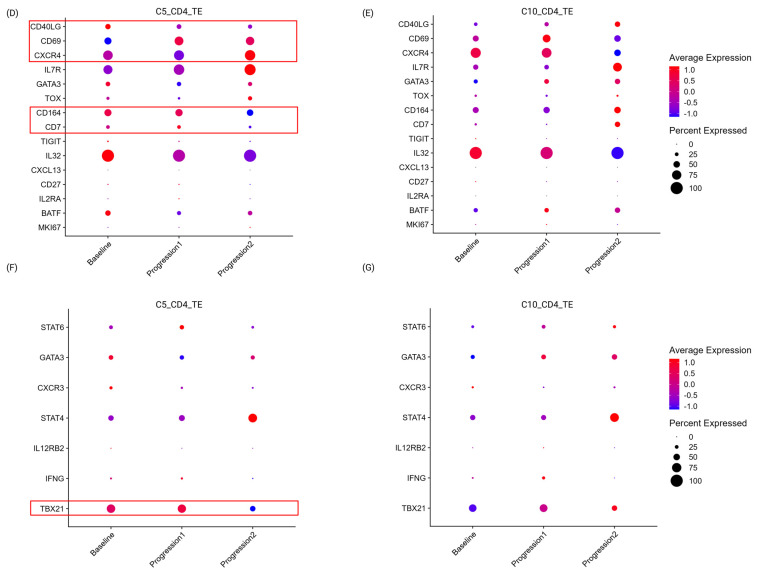
Differentially regulated genes (DEGs), associated with progression of MF. Key genes associated with C5_CD4_TE are marked with red boxes. (**A**) Dot plot of top 10 DEGs associated with progression in C5_ CD4_TE. (**B**) Dot plot of top 10 DEGs associated with progression in C10_ CD4_TE. (**C**) Top 10 unique genes associated with progression in C5_ CD4_TE. Targeted analysis of CTCL-related markers in (**D**) C5_ CD4_TE and (**E**) C10_CD4_TE. Targeted analysis of Th1- and Th2-related immune markers in (**F**) C5_ CD4_TE and (**G**) C10_CD4_TE.

To understand the molecular mechanisms associated with the disease progression, the malignant C5 and C10 CD4_TE cells were followed over time. DEGs were identified, and the most significant (based on adjusted *p*-values) genes are shown in Figure 7A (C5), Figure 7B (C10), and Supplementary Materials Tables S13 and S14. At baseline, MT-ATP8 had the relative highest expression in the C5_CD4_TE cells, while genes associated with aggressiveness of disease such as NFKBIA, JUNB, DUSP1, FOS, JUN, and RPS10 were increased at progression 1. At progression 2, additional up-regulated genes included the transcription factor RUNX3 along with genes associated with resistance to treatment and/or proliferation such as ABCB1 and RORA-AS1 (see Figure 7A and Supplementary Materials Table S13). Some genes were identically regulated in C10, including the overexpression of RASGEF1B at progression 2. To complement the analysis, unique features, observed only in C5 and not significantly differentially regulated in C10 compared to non-expanded clones, were identified (Figure 7C and Supplementary Materials Table S15). In addition to the previously mentioned RUNX3 and ABCB1, unique features included genes associated with cellular functions such as proliferation and chemoresistance by kinase c eta type (PRKCH, [35]), regulation of angiogenesis by phosphodiesterase 3B (PDE3B, [36]), degradation of cytotoxic compounds, dihydropyrimidindehydrogenas (DPYD, [37]), and motility by cell migration inducing hyaluronidase 2 (CEMIP2). To our knowledge, none of the genes have been described to play a role in the progression of MF previously. One of the genes, RUNX3 has been associated with CD8 lineage and tumor suppression in MF, but somewhat unexpectedly, here, the expression was highly up-regulated at progression 2. However, recent reports show that RUNX3 may also play a role in aggressiveness of disease in solid tumors [38,39].

Selected CTCL-related genes, previously identified in the literature [30,40,41,42,43,44,45,46,47] were visualized across time in the malignant clusters C5 (Figure 7D) and C10 (Figure 7E) (see Supplementary Materials Table S16). Of note, both malignant clusters had increased levels of GATA 3, TOX, and IL7R and reduced expression of IL32 at progression 2. Unique features for the progression-related C5 cluster included increase in CD69 and CXCR4 at progression 2, and reduction in CD40LG, CD7, and CD164 at progression 2.

Furthermore, selected DEGs associated with either a T helper cell 1 or 2 profile were explored in a targeted analysis, and pinpointed the up-regulation of STAT4 in the later stage of progression along with the down-regulation of TBX21/T-bet in C5 (Figure 7F,G). The up-regulation of STAT4 is in contrast to previous studies suggesting that loss of STAT4 might be related to disease progression [48]. However, the loss of TBX21/T-bet specifically in C5 further supports the role of C5 in driving disease progression in the studied patient.

In summary, novel genes not previously associated with progression in MF were identified and included several treatment resistance-, migration-, and angiogenesis-related genes such as ABCB1, PRKCH, PDE3B, DPYD, and CEMIP2.

### 3.2. Spatially Guided Transcriptional Analysis in Tissue, Focused on Intra-Patient Heterogeneity Across Spatial Regions and Time

#### 3.2.1. QC and Number of Collected Cells in Different Segments

The number of ROIs for each phenotypic cell segment and information on their spatial location (close to, or far away from epidermis) are available in Supplementary Materials Table S17. Cell deconvolution, using SpatialDecon workflow, was performed to investigate the estimated abundance of immune cell types in the phenotypic cell segments (Supplementary Materials Figure S8). As expected, a significantly higher predicted proportion of cytotoxic CD8+ T-cells in the CD3+/CD8+ segment and a significantly higher predicted proportion of macrophages in the CD163+ segments was observed. The predicted proportion of CD4 T-cells in the CD3+/CD8− ROIs is high; however, the CD163+ and segments also have a significant predicted proportion of CD4 T-cells, probably reflecting the fact that CD4 as a molecule is highly expressed in CD163+ cells and the accuracy of the CD4 T-cell prediction may be compromised due to that.

#### 3.2.2. Intra-Patient Heterogeneity Related to Spatial Localization

**Figure 8:**
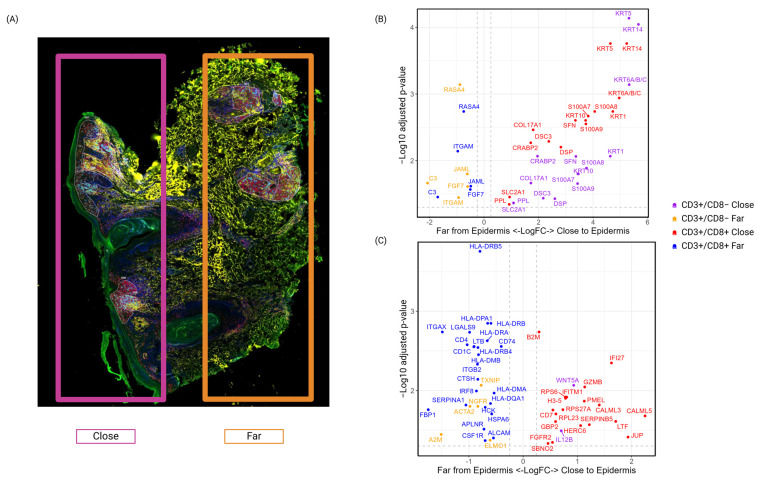
Tissue-based analysis reveals intra-tumor heterogeneity at early progression. (**A**) Different regions were defined as either close to the epidermis (pink box) or far from the epidermis (orange box) in the tissue biopsy at progression 1. (**B**) Common DEGs in CD3+/CD8− and CD3+/CD8+ cell segments when the two spatial localizations are compared. (**C**) Unique DEGs for each of the CD3+/CD8− and CD3+/CD8+ cell segments, respectively, when the same two spatial localizations are compared.

We hypothesized that the comparison of transcriptional profiles in different spatial localizations (see Figure 8A) would reveal intra-tumor variation. DEGs up-regulated in both CD4 (T-helper cells (CD3+/CD8−)) and CD8 (T-cytotoxic cells (CD3+/CD8+)) T-cell segments in regions close or distal to the epidermis, in skin biopsy from progression 1, were identified (Figure 8B, Supplementary Materials Table S18). No DEGs were found comparing spatial localizations close or distant from epidermis among CD163+ segments. T-cell-related DEGs with higher expression values close to the epidermis included a wide range of structural-related genes such as keratin, collagens, desmoplakins, and adhesion-related molecules such as desmocollin. S100 family proteins involved in cell cycle progression/differentiation and specific genes associated with invasion and metastasis (BRABP21) were also up-regulated. DEGs with higher expression values more distant from the epidermis included integrins (ITGAM1), adhesion-related molecules (JAML1), and growth factors (FGF71).

Unique DEGS in either the CD4 or CD8 T-cell segments associated with one of the two spatial regions were also identified (Figure 8C and Supplementary Materials Tables S19 and S20). We conclude that DEGs unique to the tentative malignant CD3+/CD8− cell segments were relatively few but included WNT5A [49] and IL12B, which had higher expression close to the epidermis (Figure 8C). In regions further away from the epidermis, higher expression of neural growth factor receptor (NGFR), actin alpha 2 (ACTA2), the protease inhibitor alpha-2-macroglobulin (A2M), ELMO1, and TXNIP were detected. NGF, the ligand of NGFR, is previously reported to be elevated in the affected skin of CTCL patients [50]. ELMO is associated with migration [51] and TXNIP is considered a master regulator of cellular oxidation and is activated in response to stress [52]. Based on our analysis, we cannot conclude if a proportion of malignant cells have more aggressive features in one of the two spatial localizations. We can only speculate on which functions are driven by the DEGs that differentiate the two localizations. For example, the overexpression of TXNIP far from the epidermis has been linked to the maintenance of regulatory T-cell activity, a feature that may be important for disease progression, despite that our peripheral blood-based scRNA-seq analysis do not identify such a population [53].

In cytotoxic CD3+/CD8+ cells, several HLA-DR-related genes that indicate T-cell activation [54] had higher expression far from the epidermis (Figure 8C). Of note, CD7, a marker related to naïve and memory CD8 cells, was higher in cell segments collected close to, compared to far away from, the epidermis and might indicate that CD8 T-cells in more distant regions are mostly effector CD8.

#### 3.2.3. Progression of Disease

**Figure 9:**
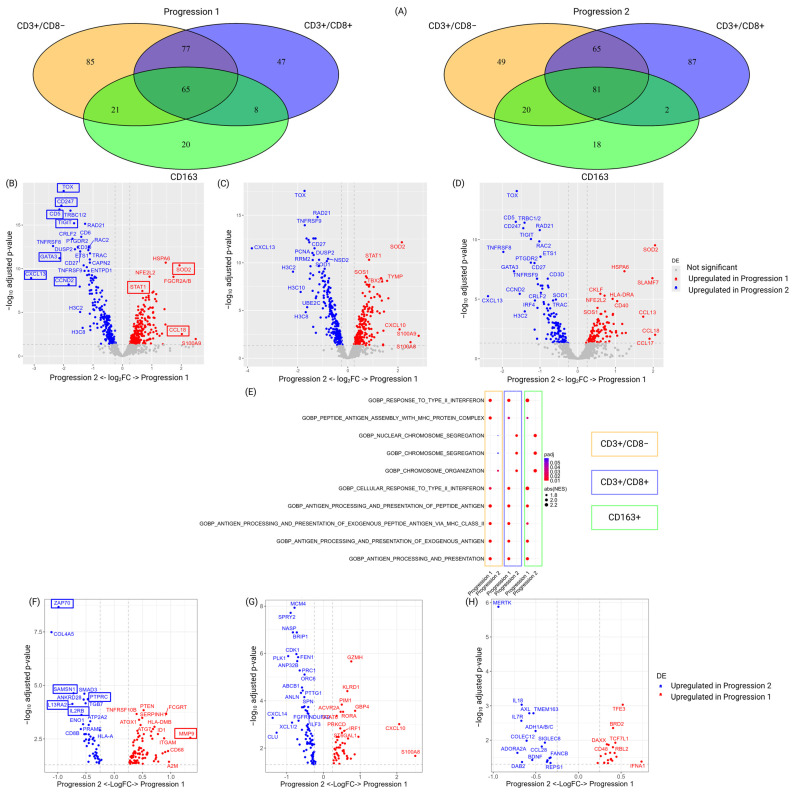
Differentially regulated genes differentiating 1st and 2nd progression. (**A**) Venn diagram showing the number of unique and common DEGs comparing progression 1 and 2 in the three cell type segments. DEGs with higher expression at progression 1 are shown to the left, and DEGs with higher expression at progression 2 are shown to the right. (**B**) Volcano plot of all DEGs identified for each of the cell segments. DEGs with higher expression at progression 1 are shown in red to the right, and DEGs with higher expression at progression 2 are shown in blue to the left for (**B**) CD3+/CD8−cell segments, (**C**) CD3+/CD8+ cell segments, and (**D**) CD163+ cell segments. (**E**) Top five identified pathways driven by the identified DEGs in targeted cells in the immune microenvironment in MF tissue cells comparing progression 1 and progression 2. Volcano plots of DEGs that are unique for each of the cell segments are shown in (**F**) for CD3+/CD8− cell segments, in (**G**) for CD3+/CD8+ cell segments, and in (**H**) for CD163+ cell segments. Genes marked in red and blue indicate features associated with the CD3+/CD8− cells.

To investigate the molecular changes associated with disease progression, CD3+/CD8− cells, CD3+/CD8+ cells, and CD163+ cells were investigated at two time points, at progression 1 and 2. Common and unique DEGs were identified for each of the three cell segment types (Figure 9A, Supplementary Materials Table S21). The different cell segments had overlapping DEGs, likely due to high expression in regions with high cell density and close cell-to-cell interactions (Figure 9B–D). Increased expression of genes at progression 1 included STAT1, SOD2, and CCL18. Increased expression of genes at progression 2 included, among others, MF-related genes such as TOX and GATA3. Also, immune regulatory and/or proliferation-related genes such as CD247, CD5, TIGIT, CXCL13, and CCND2 had higher expression in progression 2.

Pathway analysis was performed to provide a global view of cellular processes that are important for the different cell types during disease progression (Figure 9E). Antigen-related processing that included multiple Gene Ontology (GO) pathways was higher at progression 1 than 2. Processes that were up-regulated across the cell segments at progression 2 included nuclear chromosome segregation and chromosome organization (in total, three distinct GO pathways), indicating increased cell division.

Unique genes for each of the CD3+/CD8−, CD3+/CD8+, and CD163+ cell segments were visualized separately (Figure 9F–H and Supplementary Materials Tables S22–S24).

At progression 1, the tentative malignant cells included in the CD3+/CD8− cell segments had higher expression of genes associated with tissue remodeling such as MMP9. Also, PTEN, FCGRT, TNFRSF10B, ATOX1, ID1, ITGAM, CD68, and A2M had higher expression at progression 1. At progression 2, increased expression of several cell signaling-related genes was identified. These included the cytoplasmic tyrosine kinase ZAP70, which is essential for TCR signaling [55], as well as protein tyrosine phosphatase receptor type C (PTPRC/CD45) and cell-signaling-related genes such as SAM domain, SH3 domain, and nuclear localization signals (SAMSN1), the nuclear to cytoplasmic signaling molecule SMAD3, Ankyrin repeat domain 28 (ANKRD28), and the integrin ITGB7. Other differentially regulated genes included cytokines important for cell survival such as IL13RA2 [56] and IL2RB.

To investigate changes in the tumor immune microenvironment in tissue during disease progression, CD8+ T-cells neighboring the CD3+/CD8− cells were investigated (Figure 9G). At progression 1, CD3+/CD8+ cell segments had genes associated with immune regulation such as CXCL10, S100A8, granzyme H (GXMH), Killer Cell Lectin Like Receptor D1 (KLRD1), Serine/Threonine-Protein Kinase Pim-1 (PIM1), Guanylate binding protein 4 (GBP4), STAT6, IRF1, Protein Kinase C Delta (PRKCD), and the transmembrane serine/threonine kinase, ACVR2A. At progression 2, CD3+/CD8+ cell segments had increased expression of genes associated with proliferation/cell division such as Minichromosome Maintenance Complex Component 4 (MCM4), NASP, BRCA1 interacting DNA helicase 1 (BRIP1), Cyclin-dependent kinase 1 (CDK1), polo-like kinase 1 (PLK1), ANLN, ORGC6, PTTG1, drug resistance by ABCB1, and tissue transformation or migration such as SPRY2, CXCL14, and XCL1.

It is well known that polarization of macrophages into an M2-like phenotype, characterized by CD163+ cells, can impact the immune microenvironment to support disease progression. Thus, such CD163+ cell segments were investigated to pinpoint changes across time (Figure 9H). At progression 1, CD163+ cell segments had higher expression of genes involved in proliferation, such as BRD2, TCF7L1, immune signaling, CD48, IFNA1, metabolism, TFE3, apoptosis, and DAXX. At progression 2, CD163+ cell segments had DEGs such as MERTK, which has been proposed as a possible target in CTCL [57], and other immune-related molecules such as IL18, IL7R, CCL28, the signal transduction AXL Receptor Tyrosine Kinase (AXL), the transmembrane protein 163 (TMEM163), alcohol dehydrogenases (ADH1A/B/C), the scavenger receptor, ADORA2A, SIGLEC8 involved in cellular interaction and signaling, the adaptor protein, disabled-2 (DAB2), and the brain-derived neurotrophic factor (BDNF [58]). The latter may be involved in pruritus in CTCL.

In summary, difference in gene expression was identified in all segment types investigated across time. Of note, among the tentatively malignant CD3+/CD8− cells, DEGs associated with tissue remodeling were mainly found at the time of progression 1, while relative higher expression of DEGs associated with cell signaling and including key survival factor IL2RB was enriched at the time of progression 2.

#### 3.2.4. Targeted Analysis of MF- and Treatment-Related Genes

**Figure 10:**
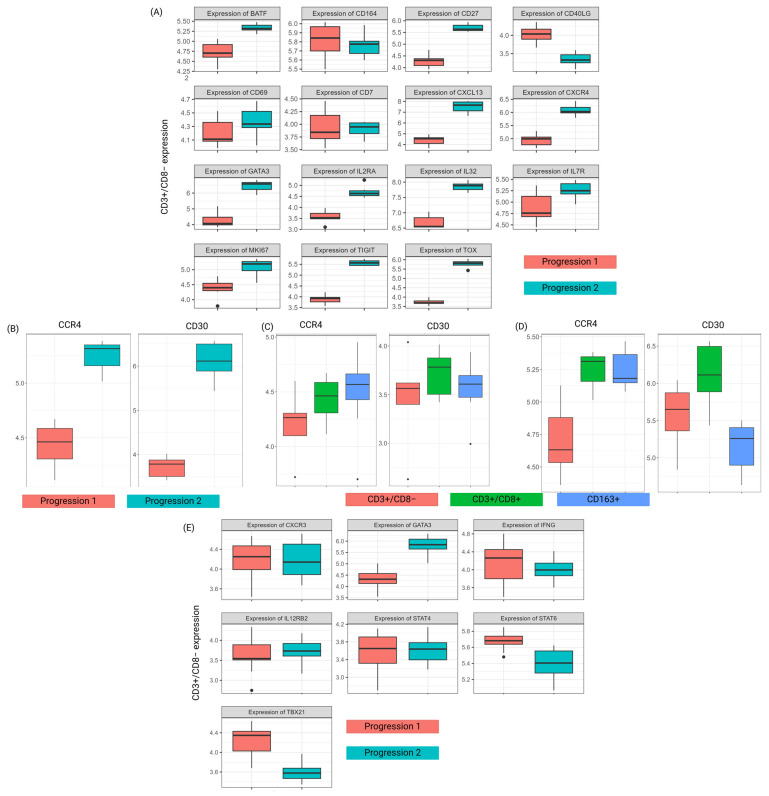
Targeted analysis. (**A**) Targeted analysis of CTCL markers (same panel as in Figure 7D,E), shown as box plots comparing progression 1 (red) and progression 2 (green) for each marker separately. (**B**) Selected genes associated with targeted CCR4 and CD30 treatment. Expression of CCR4 and CD30/TNFRSF8 across the two time points in (**B**) CD3+/CD8− cell segments. Expression of CCR4 and TNFRSF8/CD30 across the three cell segments at (**C**) progression 1 and (**D**) progression 2. (**E**) Targeted analysis of markers associated with a shift from Th1 to T2 immune profile. Comparison of expression of individual markers (same panel as in Figure 7F,G) in progression 1 (red) and progression 2 (green) in the CD3+/CD8− cell segments.

Selected MF-related genes (the same as for scRNA-seq analysis in Figure 7D,E) were investigated in the malignant CD3+/CD8− cell segments (Figure 10A). Visualization of the targeted gene list shows the relatively higher expression of TOX, GATA3, and Ki-67, but also markers such as BATF, CD27, CXCL13, CXCR4, IL2RA, IL32, IL7R, and TIGIT, at progression 2 compared to progression 1. Thus, relatively higher expression of MF-associated genes related to proliferation and activation is seen at progression 2.

To further understand how treatment-related genes CCR4 and TNFRSF8/CD30 were expressed in the different cell segments and in the two progression samples, the transcription of these genes was visualized. Both targets have higher expression at progression 2, which we interpret as a higher abundance of such cells among the analyzed CD3+/CD8− cell segments analyzed (Figure 10B–D).

Th1- and /Th2-related genes were selected and their transcription was visualized to assess if a Th1 to Th2 shift was related to disease progression. Like the C5_CD4_TE scRNA-seq cluster in peripheral blood, GATA3 had higher expression at progression 2 comparing with samples collected at the time of progression 1, while STAT6 and TBX21 had lower expression at progression 2 (Figure 10E). Similar de-regulation of Th1/Th2-related genes in the two compartments indicates that signs of progression are equally detected in tissue and peripheral blood.

## 4. Discussion

Novel advanced technologies provide means to understand intra-patient heterogeneity in MF and can also capture initiating molecular events associated with disease progression. In our study, scRNA-seq with paired TCR V(D)J sequencing and digital spatial profiling were used to characterize early events associated with disease progression. Paired samples from peripheral blood and skin derived from a patient enrolled in the BIO-MUSE clinical trial were studied. Our analysis points towards progression under treatment with mogamulizumab in the case study patient being driven by a single monoclonal T-cell population, while the total malignant population included three clones with similar gene expression patterns. Recent related publications show diverse results, with some suggesting that MF has a monoclonal origin of mature skin-homing T-cells [17] while another suggests a more oligoclonal or polyclonal nature [59]. Thus, our study contributes to the understanding of the clonal diversity in MF.

MF is believed to be derived from the skin-resident effector memory T-cell subset (TEM), in contrast to Sézary syndrome, which arises from central memory T-cells (TCM) [9]. However, others have reported that multiple distinct subsets, including naïve T-cells, TCM, TEM, and terminal effector memory T-cells (TEMRA) cells, can be identified among malignant cells in MF [60]. Here, in peripheral blood of the patient, we identify two types of expanded CD4 T-cells, terminal effector (CD4_TE) and effector memory (CD4_EM), along with two expanded CD8 populations. In line with a previous publication, few peripheral blood CD3 populations showed proliferative features, in contrast to skin biopsies [61].

In our study, gene expression analysis was combined with clonal analysis to identify the malignant clone based on increased relative frequency upon disease progression. At baseline, the malignant CD4_TE clusters (5 and 10) were fully dominated by three clones, and with similar gene expression pattern. However, only C5 increased in relative frequency during progression and is, thus, assumed to be the peripheral-blood-derived clone that drove disease progression.

Previous studies have indicated that MF may be driven by diverse genetic programs promoting either activation or proliferation-related gene signatures [62]. Also in our study, some variation in gene expression within the two malignant CD4_TE clusters that constituted three distinct TCR clones were detected. Common differences that unite the two malignant CD4_TE clusters (5 and 10) include the relatively lower expression of CD7 and higher expression of killer cell lectin like receptor B1 (KLRB1). Such features distinguished them from other expanded CD4 and CD8 clones. Down-regulation of CD7 is a known common feature of CTCL [63], and, thus, supports our assumption of a malignant phenotype for the C5 and C10_CD4_TE cells.

Of note, in a sub-analysis, we identify key features of C5_CD4_TE that were not found in any other expanded cells of either CD4 or CD8 origin. These features are not generally associated with the hypothesized expanded malignant CD4_TE cells but specifically with the peripheral-blood-derived clonal cells that are expanded during disease progression. At baseline before progression, TGFB1 and LTBP4 are specifically up-regulated in this cell cluster, indicating that progression can be initiated/driven by TGF-β-related signaling networks. Of note, a recent study investigated the possibility to target CD51 on CTCL, as such inhibition could lead to reduced TGF-β activation [64]. The role of TGF-β signaling in cancer and disease progression is well described, and the possibility to target this pathway has recently been reviewed by Liu et al. [65]. Although TGF-β inhibitors elicit an anticancer effect, the mechanisms are still not fully elucidated, and side-effects such as cardiovascular toxicity have delayed clinical implementation. However, novel iso-form-specific treatment may be a way forward to target TGF-β in cancer patients.

Furthermore, to understand the molecular mechanisms that govern transcriptional drift within the clone associated with disease progression, we identified changes over time that were unique in C5_CD4_TE. Early during progression, proliferation-related genes such as NFKBIA, JUNB, and DUSP1, also previously linked to CTCL, were dominating [66]. At progression 2, key genes such as RUNX3 and ABCB1 were up-regulated. ABCB1 is associated with treatment resistance to commonly used agents in dermatology such as methotrexate, topical steroids, cyclosporine, and biological agents, as recently reviewed [67]. Of interest, RUNX3 is associated with the acquisition of cytotoxic features by CD4 T-cells [68], indicating that such a transition is associated with the disease progression in this specific patient.

A targeted analysis of 15 CTCL-related genes was performed to study disease progression and included migration/adhesion-related molecules such as CD69 and CXCR4. The relative up-regulation during disease progression of these molecules in the progression-related C5 cluster is consistent with the migratory properties of the malignant cells. Similarly, target analysis of seven genes previously known to be involved transition from aTh1 to aTh2 immune profile was used to evaluate a tentative shift among the malignant clones. Of major interest, down-regulation of TBX21/T-bet during disease progression was observed in C5_CD4_TE but not in C10_CD4_TE, emphasizing that that the shift in the malignant cells from Th1 to Th2 profile may be more prominent in the progression-related clone compared to the other malignant cells. Also in skin biopsies, down-regulation of TBX21/T-bet and up-regulation of GATA-3 was seen during disease progression in the CD3+/CD8− cell segment, supporting the hypothesis that parallel adaptation takes place in blood and tissue. In tissue, down-regulation of STAT6 during disease progression was also seen, consistent with the change from a Th1- to Th2-related immune profile.

We also found non-malignant expanded clones of both CD4 and CD8 origin. Clonal inflammatory T-cells may derive from several different immunological process, and can be related to the common bacterial infections seen in the case study patient and other patients with MF, as recently discussed by Gaydosik et al. [29]. Gene expression profiles of such expanded non-malignant T-cell clones overlapped between our study and Gaydosik et al., but further discussion of these genes is outside the scope of the current paper.

Multiple therapeutic options are available for MF patients, and for subsets of patients, targeted antibody treatment is feasible. Several alternatives are currently used in CTCL, including CD47, C-C chemokine receptor 4 (CCR4), and CD30 [69]. Mogamulizumab targets CCR4 [19], while brentuximab–vedotin targets CD30 and releases a drug conjugate affecting microtubule function. We observed a higher expression of both CCR4 and TNFRSF8 in skin biopsies at progression 2, which may indicate that malignant cells expressing such molecules are specifically expanded during disease progression.

Using skin biopsies, we were also able to investigate transcriptional differences related to the spatial localization of sampled cell segments. Our analysis shows that CD3+/CD8− cell segments distant from the epidermis, as compared with cell segments close to the epidermis, show features of migration (ELMO1) and cellular stress (TXNIP). In CD8+ T-cell segments collected distant, compared to close, to the epidermis, cell segments seemed to be more activated and with up-regulated HLA-DR-related molecules.

TOX and GATA-3, known MF-related genes, were up-regulated with progression, and emphasize that malignant cells are enriched in the skin as MF progress. Unique DEGs that increased with progression in the tentative malignant CD3+/CD8− cell segments included a wide range of cell signaling molecules, such as ZAP70, CD45, SAMSN1, SMAD3, and ANKRD28. In addition, key proliferation markers were up-regulated, and pathway analysis supported an increased use of genes associated with chromosome segregation. Overall, CD3+/CD8− cell segments showed more pronounced malignant features across time.

Several limitations are associated with this study, with the most prominent being the fact that conclusions can only be made for the specific case study patient and that additional time points would have provided a better time-related resolution. However, we conclude that as MF is highly heterogenous, single patient studies also add to the scientific knowledge about the variety of factors that drive disease progression. However, findings need to be validated in retrospective cohorts of patients. Also, a proportion of cells in the scRNA-seq analysis were labeled as double negative, lacking markers for both CD4 and CD8 lineage, which limited further analysis of those cells. On the biological level, inclusion of additional immune subtypes in both the scRNA-seq and spatial omic studies could have provided additional information; for example, B-cells have recently been identified as an important player in CTCL [70], but such aspects are not covered by the specific study.

## 5. Conclusions

In summary, here, we advance the understanding of disease progression in MF through the analysis of a case study BIO-MUSE patient that allowed the identification of a clonotype specifically associated with disease progression. Technology advances allow parallel measurement of a wide set of molecular features in patient samples of different origin, such as peripheral blood and skin biopsies studied here. Our study contributes to the understanding of the clonal diversity in MF, and we conclude that oligoclonality may precede later progression driven by a single clone. With the focus on Th1/Th2-related molecules, we conclude that such a shift in the malignant cells is detected at the same time point in peripheral blood and skin biopsies. Of note, when focusing our analysis to specific features associated with the progression-related C5_CD4_TE clone, we detect specific up-regulation of TGF- β-related molecules prior to progression. Thus, we suggest that analyses of TGF- β in larger MF cohorts should be conducted to understand how frequent elevated levels precede progression.

## Figures and Tables

**Table 1:** Clinicopathological parameters for the case study patient across the three samples collected at baseline, progression 1, and progression 2.

Clinical Parameter	Baseline	Progression 1	Progression 2
TNMB ^a^	T_1_N_0_M_0_B_1b_	T_3_N_0_M_0_B_1_	T_3_N_0_M_N/A_B_0a_
Stadium	IA	IIIB	IIB
mSWAT ^b^	6.5	14	13
LDH ^c^	3.1	3.8	4.8
Treatment modality	Mogamulizumab + Dermovat	Mogamulizumab + Dermovat	Brentuximab vedotin and CHEP
Site of biopsy	Left flank	Neck	Right chest

^a^ Tumor node visceral blood; ^b^ modified Severity-Weighted Assessment Tool; ^c^ lactate dehydrogenase.

**Table 2:** Annotation and naming of the nine different cell types identified using semi-automated labeling of the identified 16 cell clusters derived from the UMAP analysis.

Cluster ID	Cell Type Description	Cell Cluster Abbreviation
0	Double negative T-cells	C0_Double negative
1	Non vd2 gd T-cells	C1_Non-vd2 gd
2	CD8 effector memory T-cells	C2_CD8_EM
3	CD8 effector memory T-cells	C3_CD8_EM
4	CD8 terminal effector T-cells	C4_CD8_TE
5	CD4 terminal effector T-cells	C5_CD4_TE
6	CD4 effector memory T-cells	C6_CD4_EM
7	Th1/Th17 cells	C7_CD4_Th1/Th17
8	CD8 terminal effector T-cells	C8_CD8_TE
9	CD4 effector memory T-cells	C9_CD4_EM
10	CD4 terminal effector T-cells	C10_CD4_TE
11	CD4 effector memory T-cells	C11_CD4_EM
12	CD4 naïve/central memory T-cells	C12_CD4_naive_CM
13	Non vd2 gd T-cells	C13_Non-vd2 gd
14	Proliferating effector T-cells	C14_Prolif_E
15	CD4 naïve/central memory T-cells	C15_CD4_naive_CM

## Data Availability

The datasets presented in this article are not readily available because the data are part of an ongoing clinical study. Requests to access the datasets should be directed to sara.ek@immun.lth.se.

## Supplementary Materials

The following supporting information can be downloaded at: https://www.mdpi.com/article/10.3390/biomedicines13010186/s1.
