# Supplementary material for: The Progression of Mycosis Fungoides During Treatment with Mogamulizumab: A BIO-MUSE Case Study of the Tumor and Immune Response in Peripheral Blood and Tissue
Source: Biomedicines. 2025 Jan 14;13(1):186. doi: 10.3390/biomedicines13010186 (PMC11761615; doi:10.3390/biomedicines13010186)
Supplement: Supplementary file 1 [file biomedicines-13-00186-s001.zip › Supplementary Materials Figures.pdf]

Supplementary figures

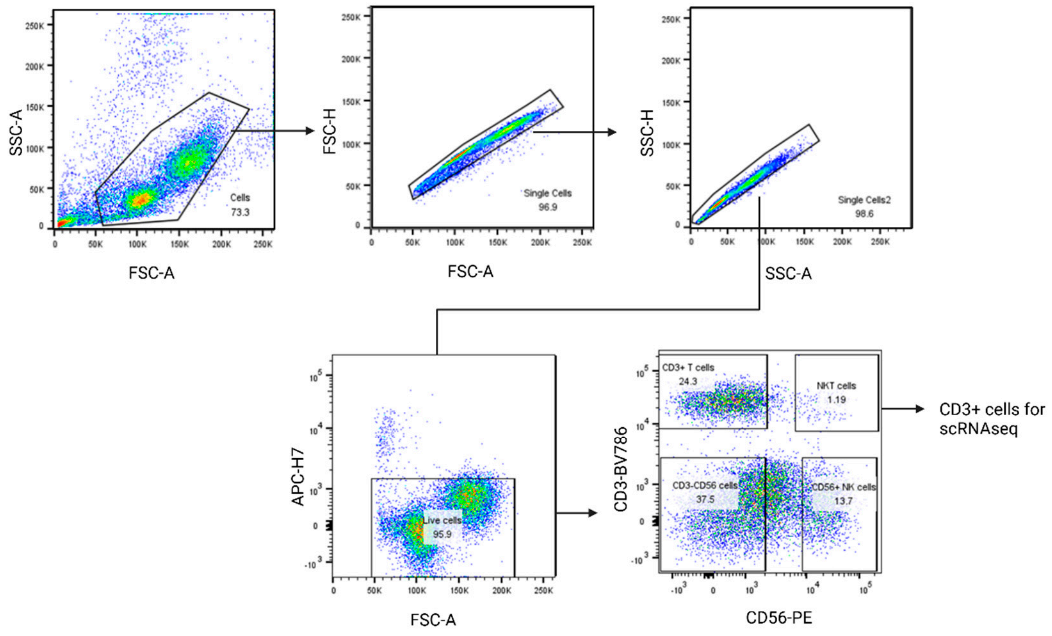

Figure S1: **Representative gating strategy** of the flow cytometry sorting of CD3+ cells used in the scRNAseq. Forward scatter (FSC) versus Side scatter (SSC) area was used to exclude debris and discriminate cells according to relative size and granularity. Doublets were removed using FSC-A Vs. FSC-H followed by SSC-A Vs. SSC-H parameters. A viability stain (APC-H7) was used to discriminate dead cells before gating on the CD3+ cells that was sorted and used for scRNAseq.

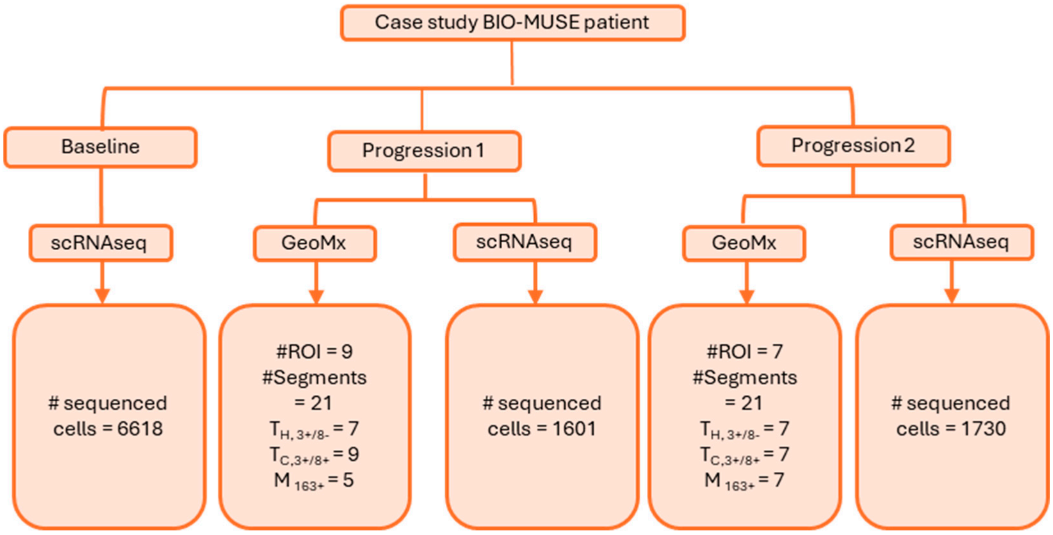

Figure S2: **Information overview for the case study sample**. The summative figure includes timepoints, type of technology, number of cells for scRNA-seq analysis and number of cell type specific AOIs for GeoMx analysis.

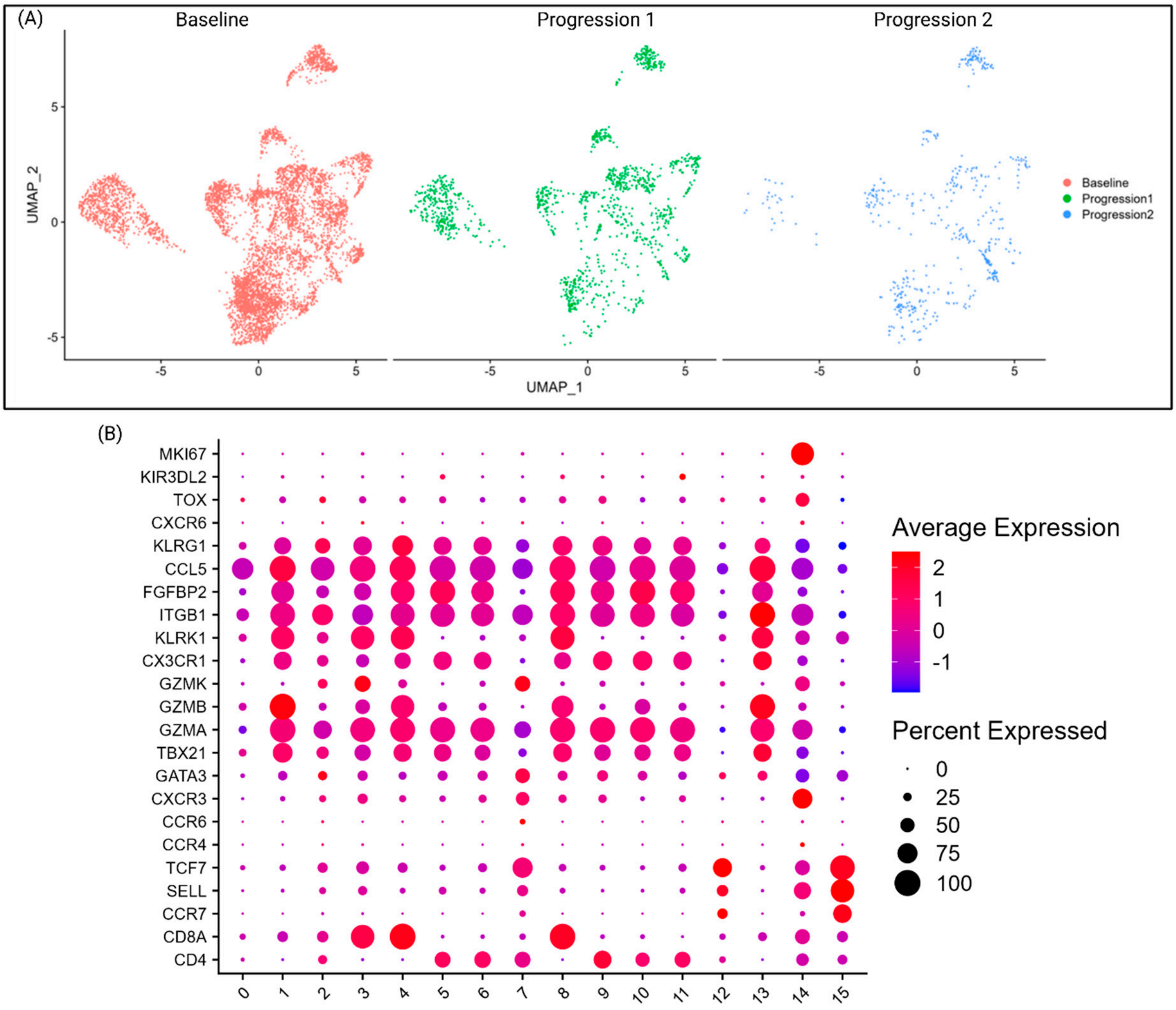

Figure S3: Overview of CD3+ T cell populations identified in the scRNA-seq analysis. A) UMAP of all sequenced cells in the three separate timepoints. B) Feature plot with relative expression of the canonical markers used for the manual cell annotation of each of the identified UMAP cell clusters.

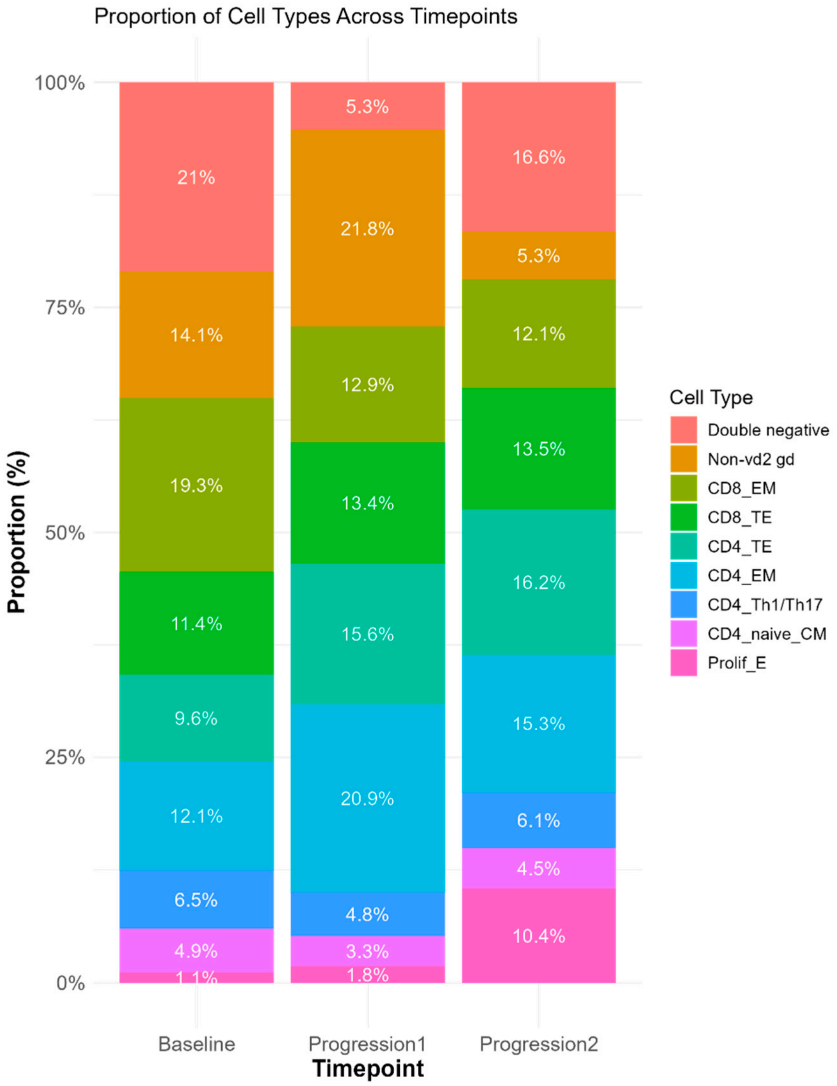

Figure S4: Cell type distribution across baseline and time points of progression of disease. Boxplots show the proportion of the different cell types at the three timepoints.

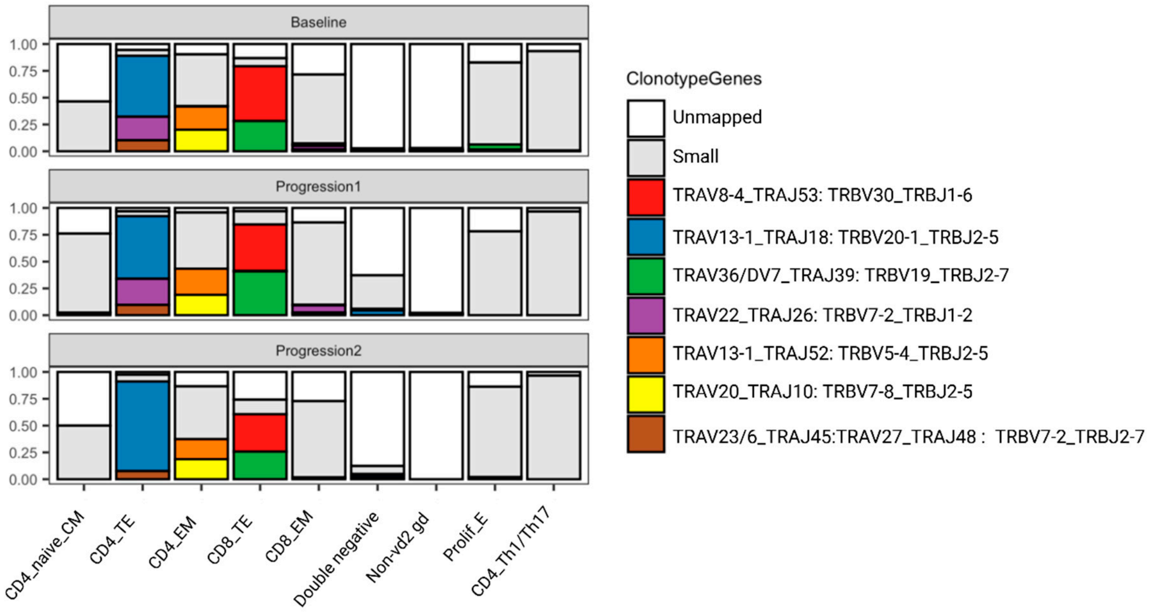

Figure S5: Clonotype distribution in different cell types across baseline and time points of progression of disease. Boxplots showing the top 7 clonotypes distributed in the different cell types at the three different timepoints. Grey indicated the collection of clonotypes are present in a low proportion and white indicate cell not being mapped to any clonotype.

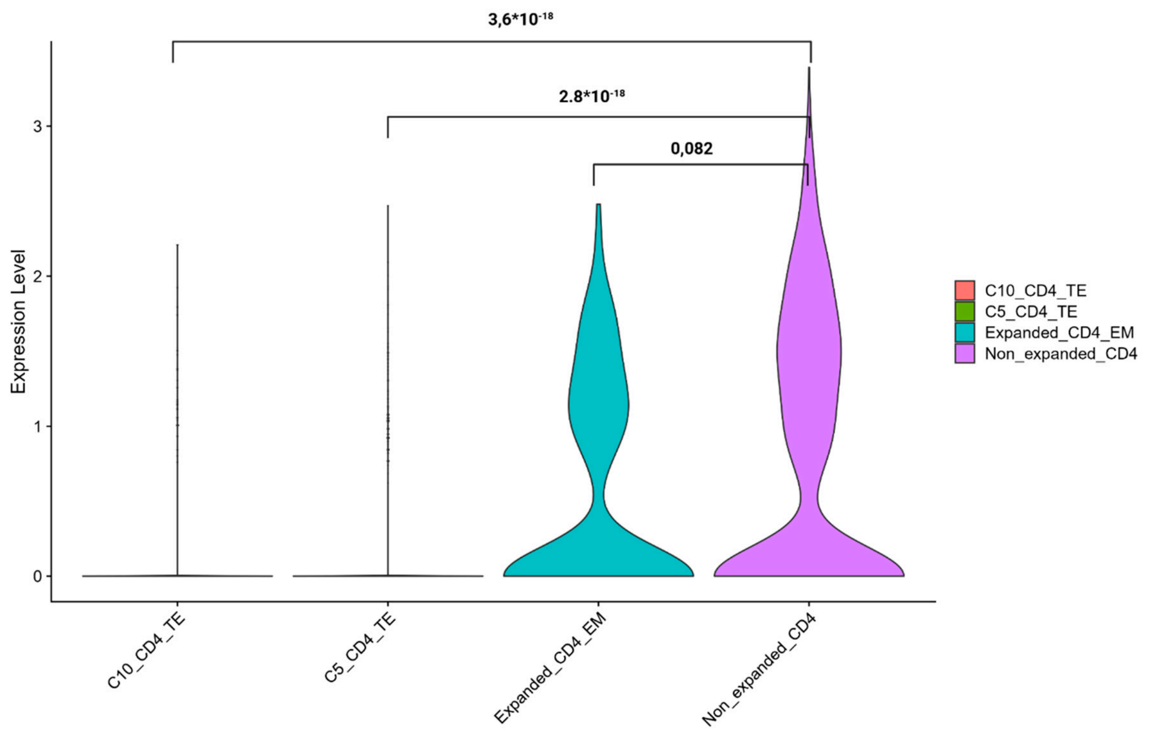

Figure S6: Expression of CD7 across different population of expanded and non-expanded CD4 T-cells. The adjusted p-value is calculated with differential expression analysis using Wilcoxon rank sum test with Bonferroni-adjusted P value.

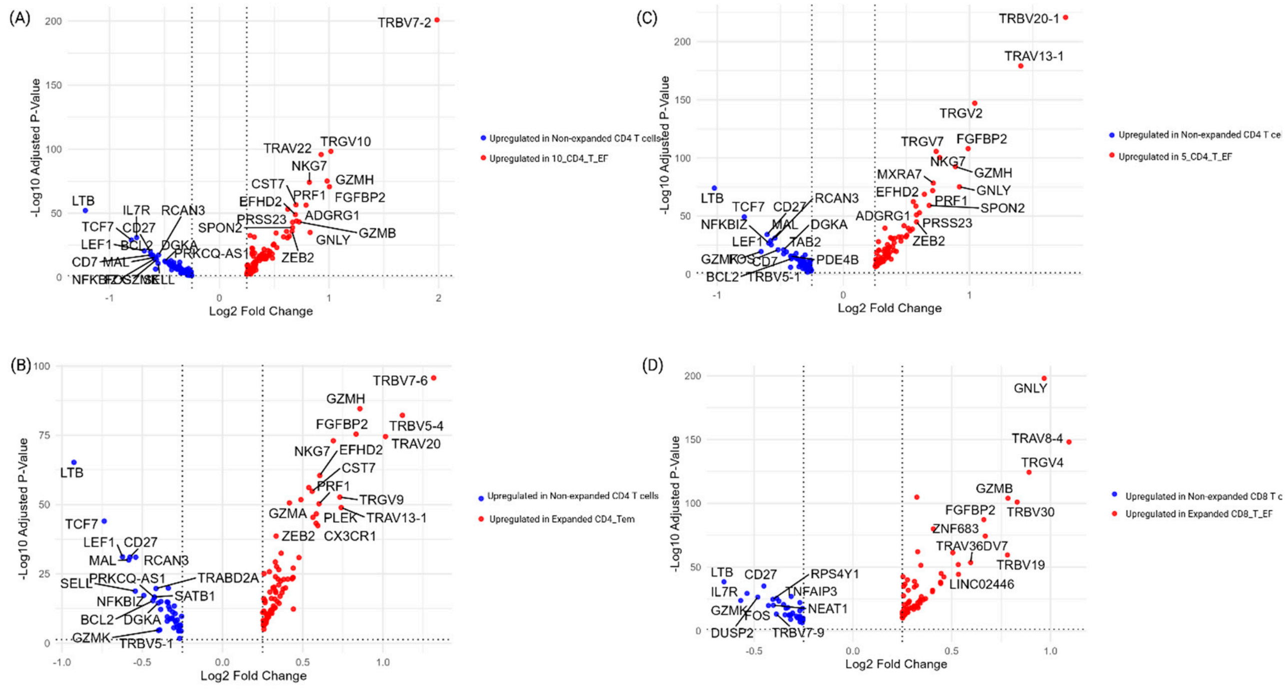

Figure S7. Volcano plots visualizing DEGs identified upon comparison of expanded and non-expanded T-cells. Volcano plots showing the differential gene expression for comparison between expanded versus non-expanded T-cells. -log10 FDR (false discovery rate) adjusted p-value was plotted against the log2 FC (fold change). The FDR adjusted p-value = 0.05 and Log2 FC=0.25 was set as cut-off. Blue color indicates genes downregulated in expanded T cells, hence upregulated in non-expanded T cells. A) Cluster C10_CD4_TE is compared to non-expanded CD4 T cells (C6, C7, C12 and C15). B) Expanded CD4_EM (C9 and C11) is compared to non-expanded CD4 T cells (C6, C7, C12 and C15). C) C5_CD4_TE is compared to non-expanded CD4 T cells (C6, C7, C12 and C15). D) Expanded CD8_TE cells (C4 and C8) are compared to non-expanded CD8 T cells (C2 and C3).

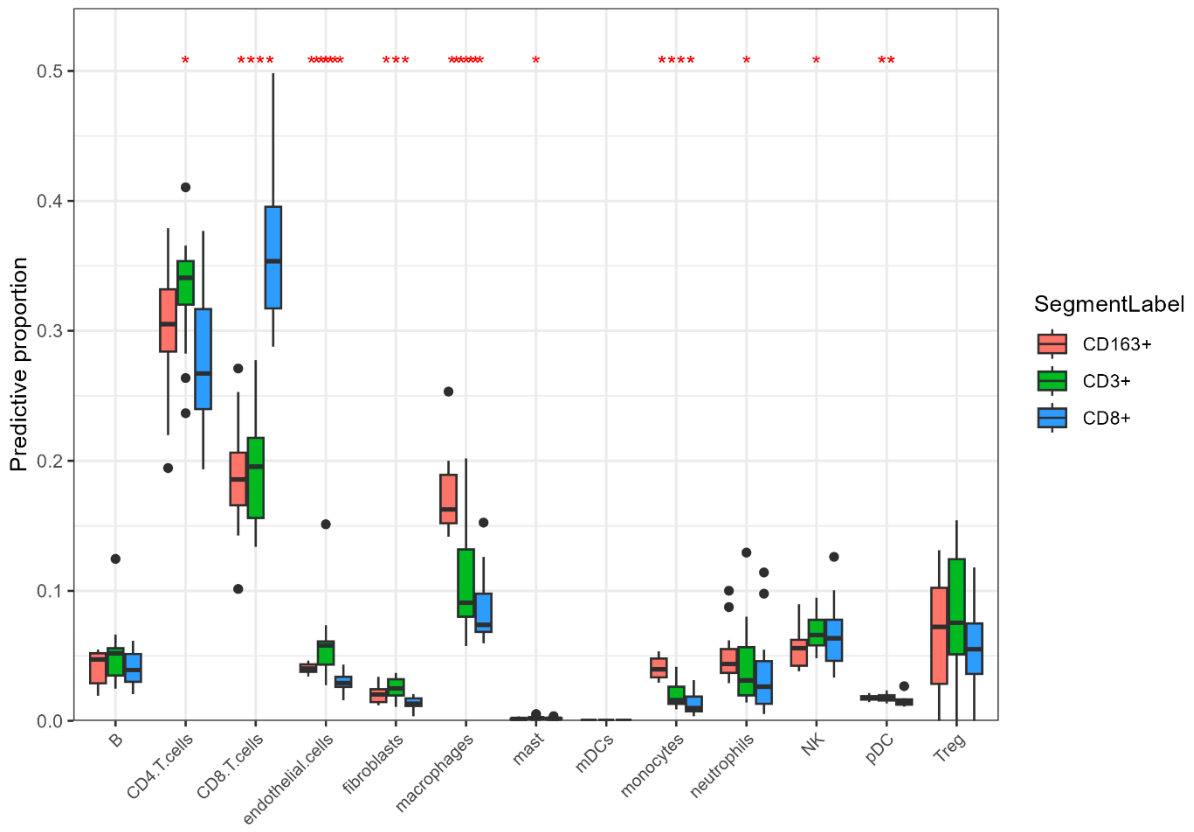

Figure S8. Cell deconvolution. Boxplot visualizing the predictive proportion using the cell deconvolution tool SpatialDecon in R. Red boxplots represents the CD163+ segments, green represents the CD3+/CD8- segments and blue represents the CD3+/CD8+ segments.
